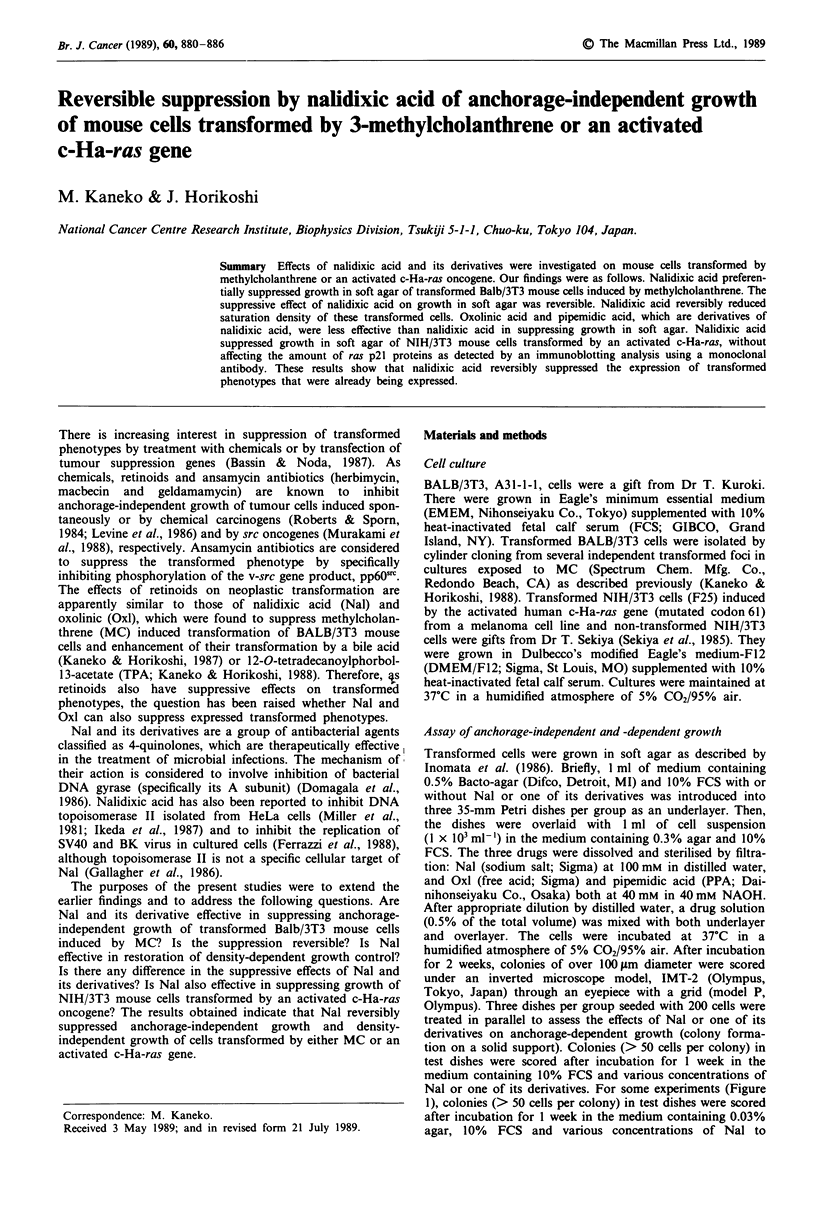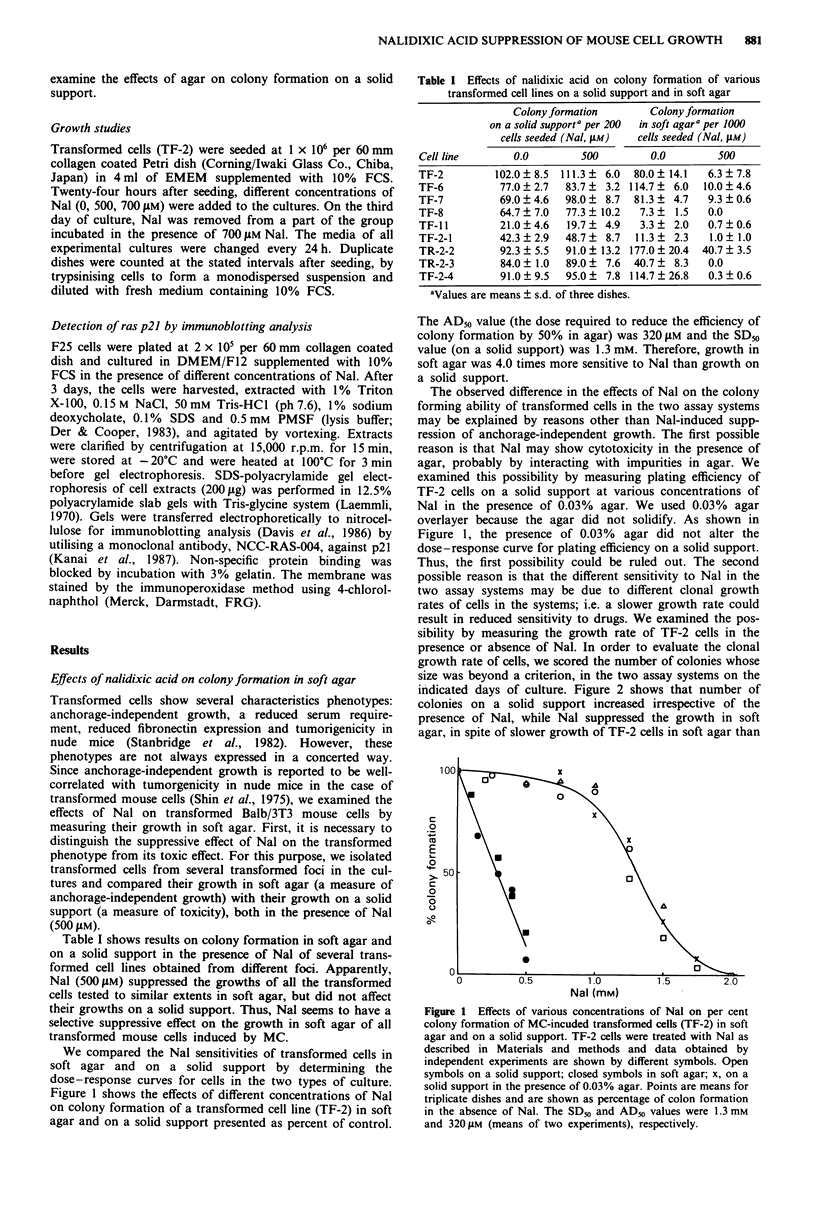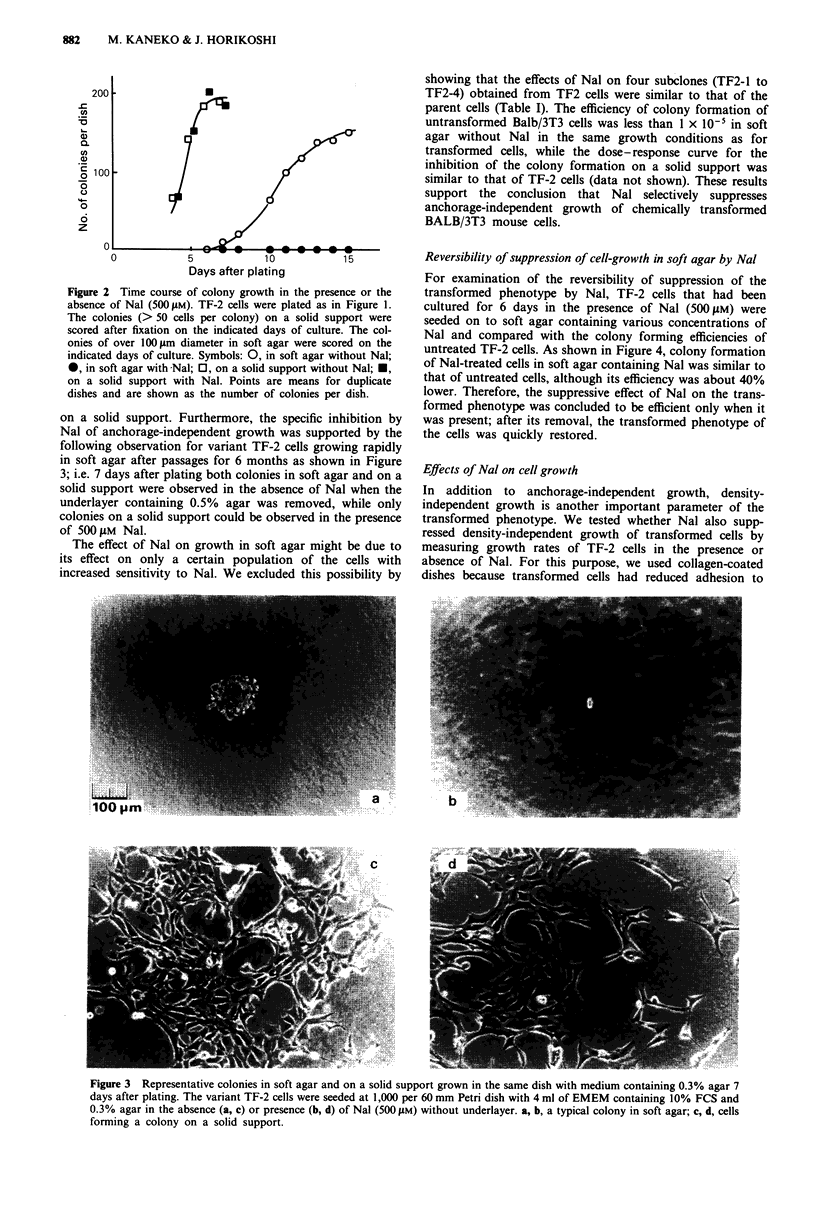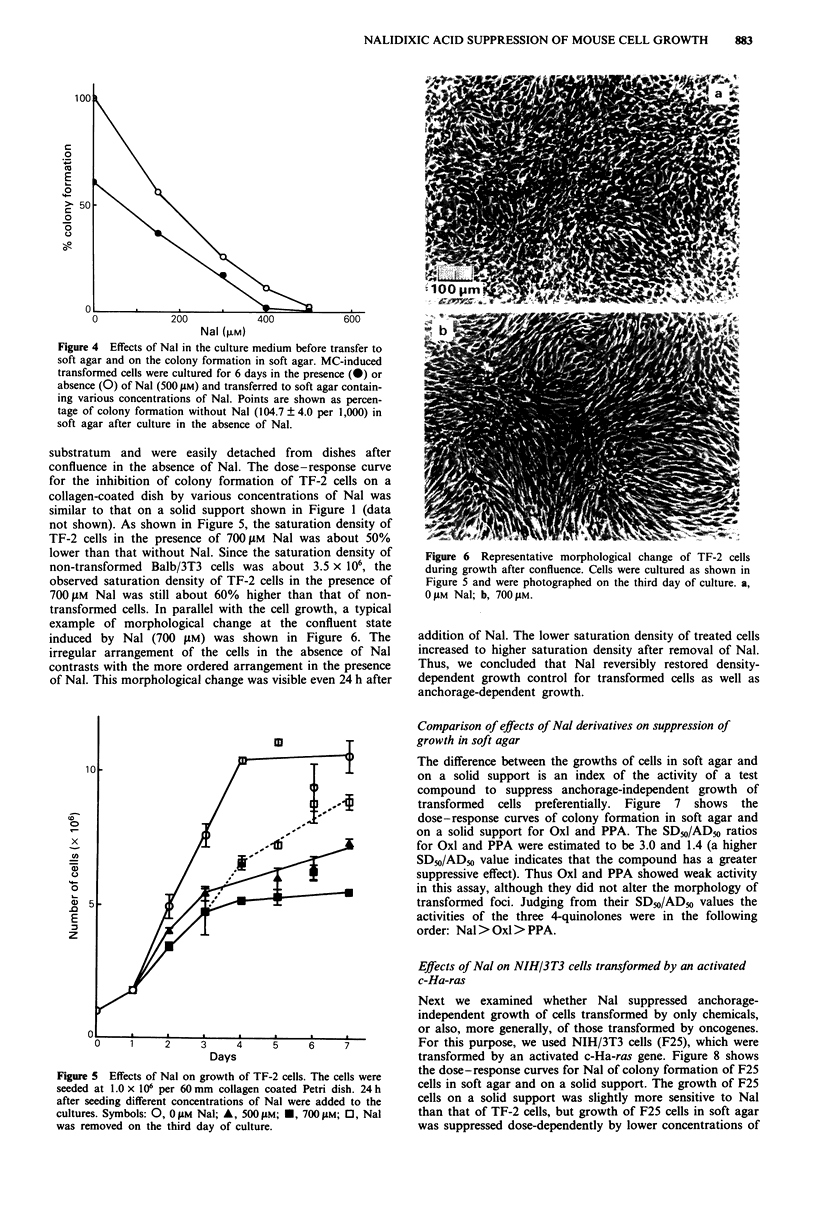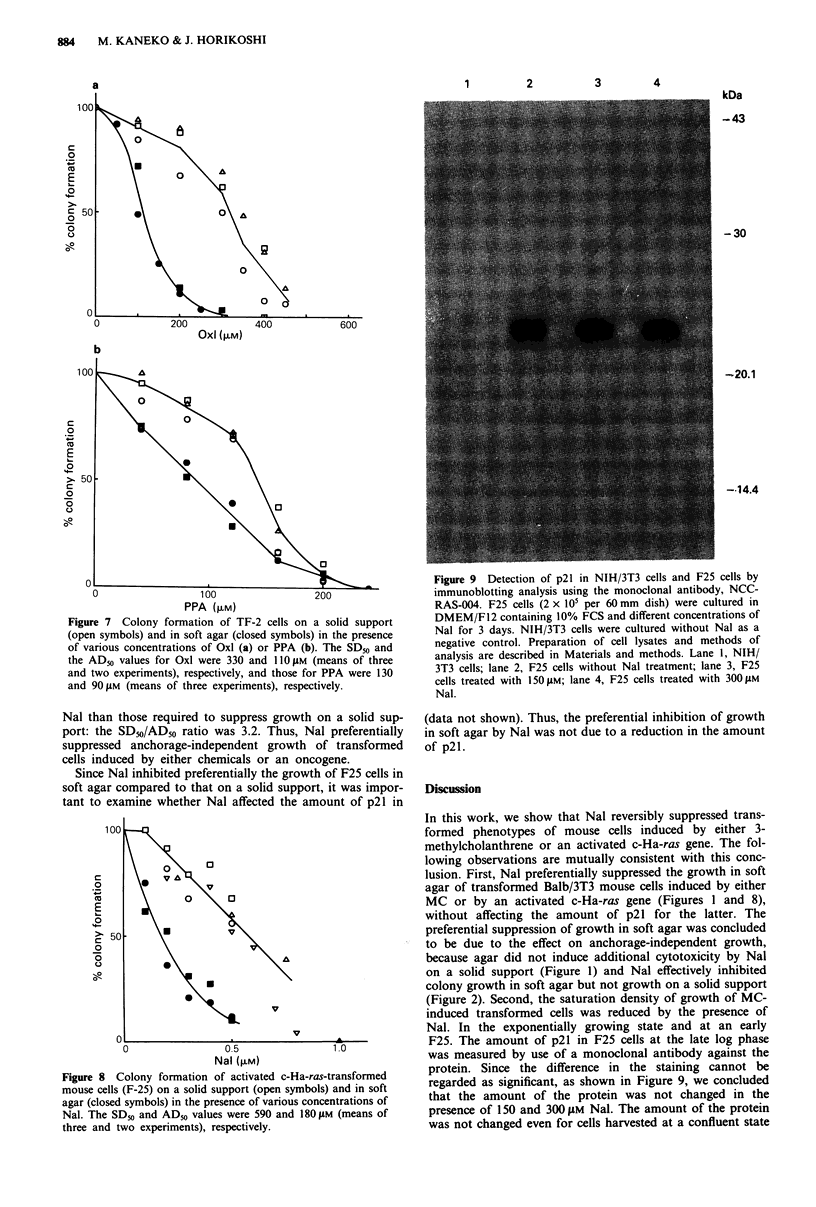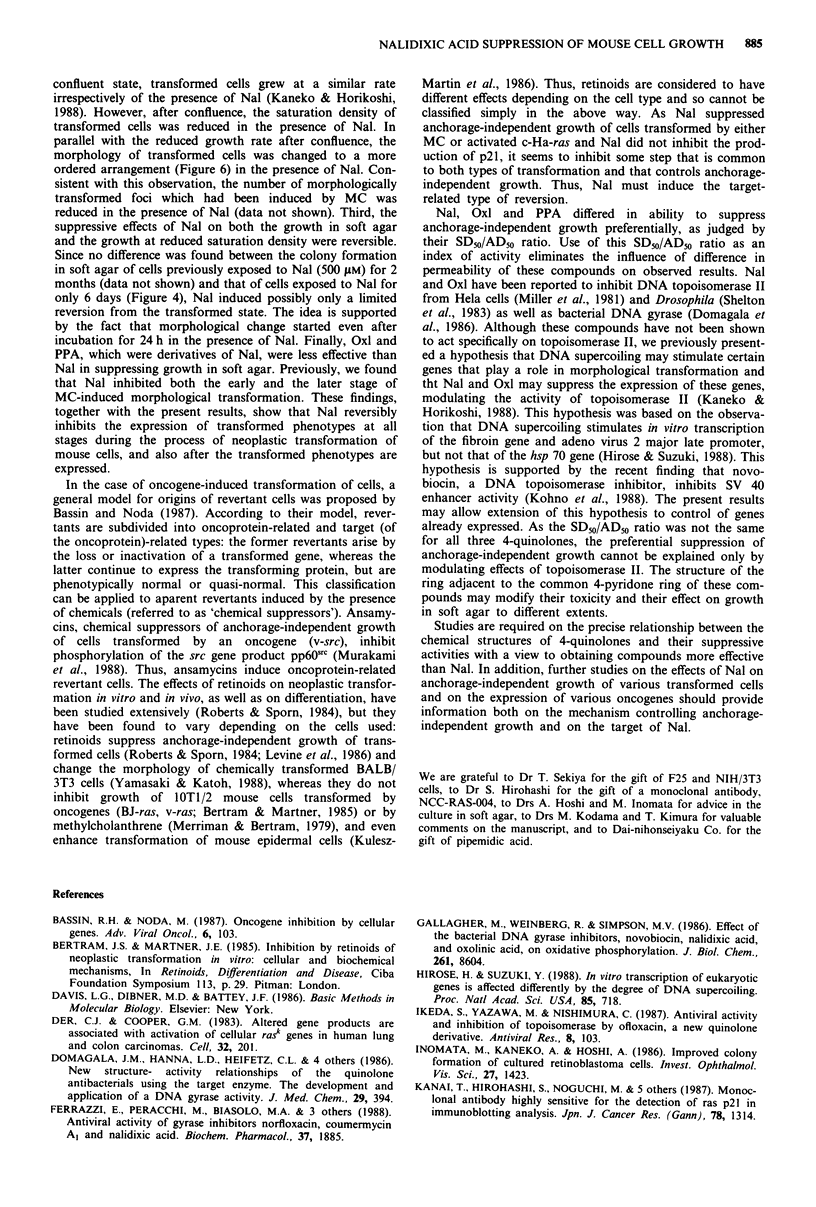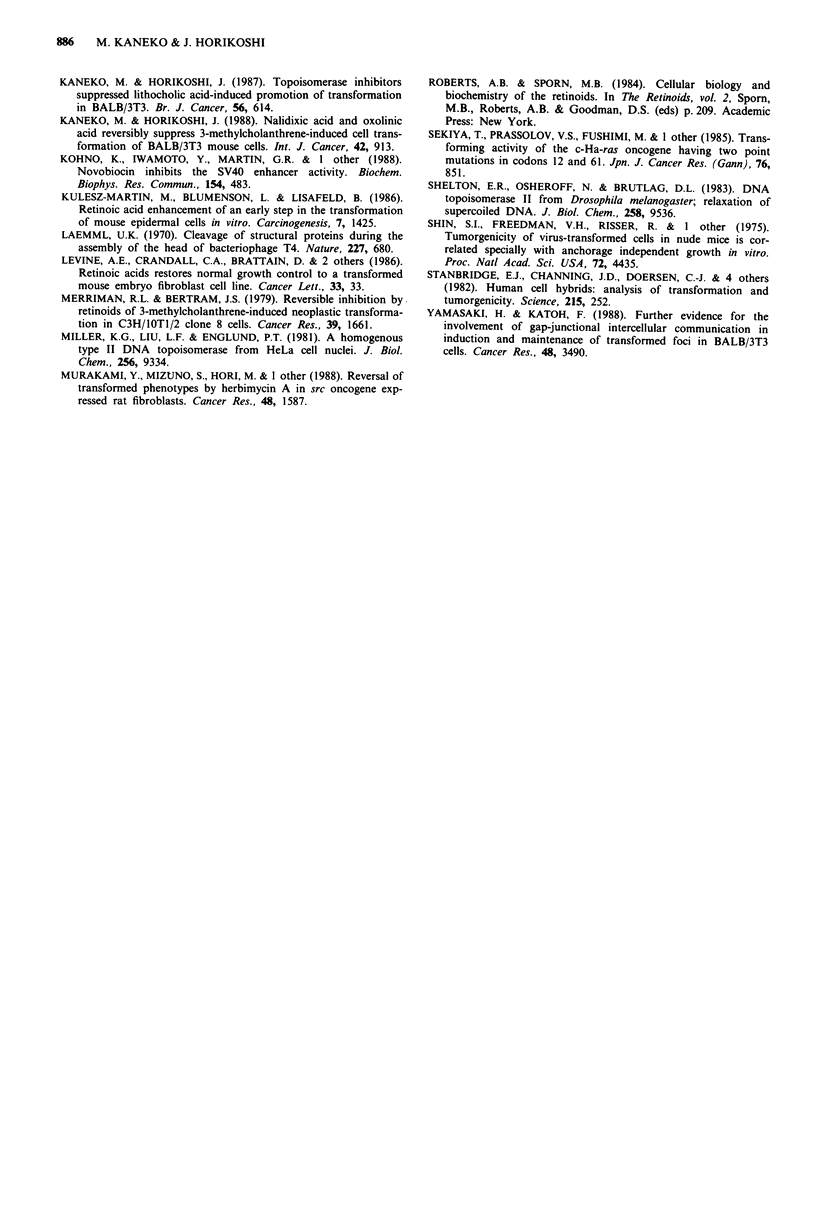# Reversible suppression by nalidixic acid of anchorage-independent growth of mouse cells transformed by 3-methylcholanthrene or an activated c-Ha-ras gene.

**DOI:** 10.1038/bjc.1989.384

**Published:** 1989-12

**Authors:** M. Kaneko, J. Horikoshi

**Affiliations:** National Cancer Centre Research Institute, Biophysics Division, Tokyo, Japan.

## Abstract

**Images:**


*Br. J. Cancer* (1989), **60**, 880–886 © The Macmillan Press Ltd., 1989

### Reversible suppression by nalidixic acid of anchorage-independent growth of mouse cells transformed by 3-methylcholanthrene or an activated c-Ha-*ras* gene

M. Kaneko & J. Horikoshi

*National Cancer Centre Research Institute, Biophysics Division, Tsukiji 5-1-1, Chuo-ku, Tokyo 104, Japan.*

**Summary** Effects of nalidixic acid and its derivatives were investigated on mouse cells transformed by methylcholanthrene or an activated c-Ha-*ras* oncogene. Our findings were as follows. Nalidixic acid preferentially suppressed growth in soft agar of transformed Balb/3T3 mouse cells induced by methylcholanthrene. The suppressive effect of nalidixic acid on growth in soft agar was reversible. Nalidixic acid reversibly reduced saturation density of these transformed cells. Oxolinic acid and pipemidic acid, which are derivatives of nalidixic acid, were less effective than nalidixic acid in suppressing growth in soft agar. Nalidixic acid suppressed growth in soft agar of NIH/3T3 mouse cells transformed by an activated c-Ha-*ras*, without affecting the amount of *ras* p21 proteins as detected by an immunoblotting analysis using a monoclonal antibody. These results show that nalidixic acid reversibly suppressed the expression of transformed phenotypes that were already being expressed.

There is increasing interest in suppression of transformed phenotypes by treatment with chemicals or by transfection of tumour suppression genes (Bassin & Noda, 1987). As chemicals, retinoids and ansamycin antibiotics (herbimycin, macbecin and geldamamycin) are known to inhibit anchorage-independent growth of tumour cells induced spontaneously or by chemical carcinogens (Roberts & Sporn, 1984; Levine *et al.*, 1986) and by *src* oncogenes (Murakami *et al.*, 1988), respectively. Ansamycin antibiotics are considered to suppress the transformed phenotype by specifically inhibiting phosphorylation of the v-*src* gene product, $pp60^{src}$. The effects of retinoids on neoplastic transformation are apparently similar to those of nalidixic acid (Nal) and oxolinic (Oxl), which were found to suppress methylcholanthrene (MC) induced transformation of BALB/3T3 mouse cells and enhancement of their transformation by a bile acid (Kaneko & Horikoshi, 1987) or 12-*O*-tetradecanoylphorbol-13-acetate (TPA; Kaneko & Horikoshi, 1988). Therefore, as retinoids also have suppressive effects on transformed phenotypes, the question has been raised whether Nal and Oxl can also suppress expressed transformed phenotypes.

Nal and its derivatives are a group of antibacterial agents classified as 4-quinolones, which are therapeutically effective in the treatment of microbial infections. The mechanism of their action is considered to involve inhibition of bacterial DNA gyrase (specifically its A subunit) (Domagala *et al.*, 1986). Nalidixic acid has also been reported to inhibit DNA topoisomerase II isolated from HeLa cells (Miller *et al.*, 1981; Ikeda *et al.*, 1987) and to inhibit the replication of SV40 and BK virus in cultured cells (Ferrazzi *et al.*, 1988), although topoisomerase II is not a specific cellular target of Nal (Gallagher *et al.*, 1986).

The purposes of the present studies were to extend the earlier findings and to address the following questions. Are Nal and its derivative effective in suppressing anchorage-independent growth of transformed Balb/3T3 mouse cells induced by MC? Is the suppression reversible? Is Nal effective in restoration of density-dependent growth control? Is there any difference in the suppressive effects of Nal and its derivatives? Is Nal also effective in suppressing growth of NIH/3T3 mouse cells transformed by an activated c-Ha-*ras* oncogene? The results obtained indicate that Nal reversibly suppressed anchorage-independent growth and density-independent growth of cells transformed by either MC or an activated c-Ha-*ras* gene.

Correspondence: M. Kaneko.
Received 3 May 1989; and in revised form 21 July 1989.

### Materials and methods

### *Cell culture*

BALB/3T3, A31-1-1, cells were a gift from Dr T. Kuroki. There were grown in Eagle's minimum essential medium (EMEM, Nihonseiyaku Co., Tokyo) supplemented with 10% heat-inactivated fetal calf serum (FCS; GIBCO, Grand Island, NY). Transformed BALB/3T3 cells were isolated by cylinder cloning from several independent transformed foci in cultures exposed to MC (Spectrum Chem. Mfg. Co., Redondo Beach, CA) as described previously (Kaneko & Horikoshi, 1988). Transformed NIH/3T3 cells (F25) induced by the activated human c-Ha-*ras* gene (mutated codon 61) from a melanoma cell line and non-transformed NIH/3T3 cells were gifts from Dr T. Sekiya (Sekiya *et al.*, 1985). They were grown in Dulbecco's modified Eagle's medium-F12 (DMEM/F12; Sigma, St Louis, MO) supplemented with 10% heat-inactivated fetal calf serum. Cultures were maintained at 37°C in a humidified atmosphere of 5% $CO_2$/95% air.

### *Assay of anchorage-independent and -dependent growth*

Transformed cells were grown in soft agar as described by Inomata *et al.* (1986). Briefly, 1 ml of medium containing 0.5% Bacto-agar (Difco, Detroit, MI) and 10% FCS with or without Nal or one of its derivatives was introduced into three 35-mm Petri dishes per group as an underlayer. Then, the dishes were overlaid with 1 ml of cell suspension ($1 \times 10^3$ $ml^{-1}$) in the medium containing 0.3% agar and 10% FCS. The three drugs were dissolved and sterilised by filtration: Nal (sodium salt; Sigma) at 100 mM in distilled water, and Oxl (free acid; Sigma) and pipemidic acid (PPA; Dainihonseiyaku Co., Osaka) both at 40 mM in 40 mM NAOH. After appropriate dilution by distilled water, a drug solution (0.5% of the total volume) was mixed with both underlayer and overlayer. The cells were incubated at 37°C in a humidified atmosphere of 5% $CO_2$/95% air. After incubation for 2 weeks, colonies of over 100 μm diameter were scored under an inverted microscope model, IMT-2 (Olympus, Tokyo, Japan) through an eyepiece with a grid (model P, Olympus). Three dishes per group seeded with 200 cells were treated in parallel to assess the effects of Nal or one of its derivatives on anchorage-dependent growth (colony formation on a solid support). Colonies (> 50 cells per colony) in test dishes were scored after incubation for 1 week in the medium containing 10% FCS and various concentrations of Nal or one of its derivatives. For some experiments (Figure 1), colonies (> 50 cells per colony) in test dishes were scored after incubation for 1 week in the medium containing 0.03% agar, 10% FCS and various concentrations of Nal to examine the effects of agar on colony formation on a solid support.

*Growth studies*

Transformed cells (TF-2) were seeded at $1 \times 10^6$ per 60 mm collagen coated Petri dish (Corning/Iwaki Glass Co., Chiba, Japan) in 4 ml of EMEM supplemented with 10% FCS. Twenty-four hours after seeding, different concentrations of Nal (0, 500, 700 μM) were added to the cultures. On the third day of culture, Nal was removed from a part of the group incubated in the presence of 700 μM Nal. The media of all experimental cultures were changed every 24 h. Duplicate dishes were counted at the stated intervals after seeding, by trypsinising cells to form a monodispersed suspension and diluted with fresh medium containing 10% FCS.

*Detection of ras p21 by immunoblotting analysis*

F25 cells were plated at $2 \times 10^5$ per 60 mm collagen coated dish and cultured in DMEM/F12 supplemented with 10% FCS in the presence of different concentrations of Nal. After 3 days, the cells were harvested, extracted with 1% Triton X-100, 0.15 M NaCl, 50 mM Tris-HCl (ph 7.6), 1% sodium deoxycholate, 0.1% SDS and 0.5 mM PMSF (lysis buffer; Der & Cooper, 1983), and agitated by vortexing. Extracts were clarified by centrifugation at 15,000 r.p.m. for 15 min, were stored at −20°C and were heated at 100°C for 3 min before gel electrophoresis. SDS-polyacrylamide gel electrophoresis of cell extracts (200 μg) was performed in 12.5% polyacrylamide slab gels with Tris-glycine system (Laemmli, 1970). Gels were transferred electrophoretically to nitrocellulose for immunoblotting analysis (Davis *et al.*, 1986) by utilising a monoclonal antibody, NCC-RAS-004, against p21 (Kanai *et al.*, 1987). Non-specific protein binding was blocked by incubation with 3% gelatin. The membrane was stained by the immunoperoxidase method using 4-chlorolnaphthol (Merck, Darmstadt, FRG).

### Results

*Effects of nalidixic acid on colony formation in soft agar*

Transformed cells show several characteristics phenotypes: anchorage-independent growth, a reduced serum requirement, reduced fibronectin expression and tumorigenicity in nude mice (Stanbridge *et al.*, 1982). However, these phenotypes are not always expressed in a concerted way. Since anchorage-independent growth is reported to be well-correlated with tumorgenicity in nude mice in the case of transformed mouse cells (Shin *et al.*, 1975), we examined the effects of Nal on transformed Balb/3T3 mouse cells by measuring their growth in soft agar. First, it is necessary to distinguish the suppressive effect of Nal on the transformed phenotype from its toxic effect. For this purpose, we isolated transformed cells from several transformed foci in the cultures and compared their growth in soft agar (a measure of anchorage-independent growth) with their growth on a solid support (a measure of toxicity), both in the presence of Nal (500 μM).

Table I shows results on colony formation in soft agar and on a solid support in the presence of Nal of several transformed cell lines obtained from different foci. Apparently, Nal (500 μM) suppressed the growths of all the transformed cells tested to similar extents in soft agar, but did not affect their growths on a solid support. Thus, Nal seems to have a selective suppressive effect on the growth in soft agar of all transformed mouse cells induced by MC.

We compared the Nal sensitivities of transformed cells in soft agar and on a solid support by determining the dose–response curves for cells in the two types of culture. Figure 1 shows the effects of different concentrations of Nal on colony formation of a transformed cell line (TF-2) in soft agar and on a solid support presented as percent of control.

**Table I** Effects of nalidixic acid on colony formation of various transformed cell lines on a solid support and in soft agar

| Cell line | *Colony formation on a solid support[a] per 200 cells seeded (Nal, μM)* | | *Colony formation in soft agar[a] per 1000 cells seeded (Nal, μM)* | |
|---|---|---|---|---|
| | *0.0* | *500* | *0.0* | *500* |
| TF-2 | 102.0 ± 8.5 | 111.3 ± 6.0 | 80.0 ± 14.1 | 6.3 ± 7.8 |
| TF-6 | 77.0 ± 2.7 | 83.7 ± 3.2 | 114.7 ± 6.0 | 10.0 ± 4.6 |
| TF-7 | 69.0 ± 4.6 | 98.0 ± 8.7 | 81.3 ± 4.7 | 9.3 ± 0.6 |
| TF-8 | 64.7 ± 7.0 | 77.3 ± 10.2 | 7.3 ± 1.5 | 0.0 |
| TF-11 | 21.0 ± 4.6 | 19.7 ± 4.9 | 3.3 ± 2.0 | 0.7 ± 0.6 |
| TF-2-1 | 42.3 ± 2.9 | 48.7 ± 8.7 | 11.3 ± 2.3 | 1.0 ± 1.0 |
| TR-2-2 | 92.3 ± 5.5 | 91.0 ± 13.2 | 177.0 ± 20.4 | 40.7 ± 3.5 |
| TR-2-3 | 84.0 ± 1.0 | 89.0 ± 7.6 | 40.7 ± 8.3 | 0.0 |
| TF-2-4 | 91.0 ± 9.5 | 95.0 ± 7.8 | 114.7 ± 26.8 | 0.3 ± 0.6 |

[a]Values are means ± s.d. of three dishes.

The $AD_{50}$ value (the dose required to reduce the efficiency of colony formation by 50% in agar) was 320 μM and the $SD_{50}$ value (on a solid support) was 1.3 mM. Therefore, growth in soft agar was 4.0 times more sensitive to Nal than growth on a solid support.

The observed difference in the effects of Nal on the colony forming ability of transformed cells in the two assay systems may be explained by reasons other than Nal-induced suppression of anchorage-independent growth. The first possible reason is that Nal may show cytotoxicity in the presence of agar, probably by interacting with impurities in agar. We examined this possibility by measuring plating efficiency of TF-2 cells on a solid support at various concentrations of Nal in the presence of 0.03% agar. We used 0.03% agar overlayer because the agar did not solidify. As shown in Figure 1, the presence of 0.03% agar did not alter the dose–response curve for plating efficiency on a solid support. Thus, the first possibility could be ruled out. The second possible reason is that the different sensitivity to Nal in the two assay systems may be due to different clonal growth rates of cells in the systems; i.e. a slower growth rate could result in reduced sensitivity to drugs. We examined the possibility by measuring the growth rate of TF-2 cells in the presence or absence of Nal. In order to evaluate the clonal growth rate of cells, we scored the number of colonies whose size was beyond a criterion, in the two assay systems on the indicated days of culture. Figure 2 shows that number of colonies on a solid support increased irrespective of the presence of Nal, while Nal suppressed the growth in soft agar, in spite of slower growth of TF-2 cells in soft agar than

**Figure 1** Effects of various concentrations of Nal on per cent colony formation of MC-incuded transformed cells (TF-2) in soft agar and on a solid support. TF-2 cells were treated with Nal as described in Materials and methods and data obtained by independent experiments are shown by different symbols. Open symbols on a solid support; closed symbols in soft agar; x, on a solid support in the presence of 0.03% agar. Points are means for triplicate dishes and are shown as percentage of colon formation in the absence of Nal. The $SD_{50}$ and $AD_{50}$ values were 1.3 mM and 320 μM (means of two experiments), respectively.

**Figure 2** Time course of colony growth in the presence or the absence of NaI (500 μM). TF-2 cells were plated as in Figure 1. The colonies (> 50 cells per colony) on a solid support were scored after fixation on the indicated days of culture. The colonies of over 100 μm diameter in soft agar were scored on the indicated days of culture. Symbols: ○, in soft agar without NaI; ●, in soft agar with NaI; □, on a solid support without NaI; ■, on a solid support with NaI. Points are means for duplicate dishes and are shown as the number of colonies per dish.

on a solid support. Furthermore, the specific inhibition by NaI of anchorage-independent growth was supported by the following observation for variant TF-2 cells growing rapidly in soft agar after passages for 6 months as shown in Figure 3; i.e. 7 days after plating both colonies in soft agar and on a solid support were observed in the absence of NaI when the underlayer containing 0.5% agar was removed, while only colonies on a solid support could be observed in the presence of 500 μM NaI.

The effect of NaI on growth in soft agar might be due to its effect on only a certain population of the cells with increased sensitivity to NaI. We excluded this possibility by showing that the effects of NaI on four subclones (TF2-1 to TF2-4) obtained from TF2 cells were similar to that of the parent cells (Table I). The efficiency of colony formation of untransformed Balb/3T3 cells was less than $1 \times 10^{-5}$ in soft agar without NaI in the same growth conditions as for transformed cells, while the dose–response curve for the inhibition of the colony formation on a solid support was similar to that of TF-2 cells (data not shown). These results support the conclusion that NaI selectively suppresses anchorage-independent growth of chemically transformed BALB/3T3 mouse cells.

### *Reversibility of suppression of cell-growth in soft agar by NaI*

For examination of the reversibility of suppression of the transformed phenotype by NaI, TF-2 cells that had been cultured for 6 days in the presence of NaI (500 μM) were seeded on to soft agar containing various concentrations of NaI and compared with the colony forming efficiencies of untreated TF-2 cells. As shown in Figure 4, colony formation of NaI-treated cells in soft agar containing NaI was similar to that of untreated cells, although its efficiency was about 40% lower. Therefore, the suppressive effect of NaI on the transformed phenotype was concluded to be efficient only when it was present; after its removal, the transformed phenotype of the cells was quickly restored.

### *Effects of NaI on cell growth*

In addition to anchorage-independent growth, density-independent growth is another important parameter of the transformed phenotype. We tested whether NaI also suppressed density-independent growth of transformed cells by measuring growth rates of TF-2 cells in the presence or absence of NaI. For this purpose, we used collagen-coated dishes because transformed cells had reduced adhesion to

**Figure 3** Representative colonies in soft agar and on a solid support grown in the same dish with medium containing 0.3% agar 7 days after plating. The variant TF-2 cells were seeded at 1,000 per 60 mm Petri dish with 4 ml of EMEM containing 10% FCS and 0.3% agar in the absence (**a**, **c**) or presence (**b**, **d**) of NaI (500 μM) without underlayer. **a**, **b**, a typical colony in soft agar; **c**, **d**, cells forming a colony on a solid support.

**Figure 4** Effects of Nal in the culture medium before transfer to soft agar and on the colony formation in soft agar. MC-induced transformed cells were cultured for 6 days in the presence (●) or absence (○) of Nal (500 μM) and transferred to soft agar containing various concentrations of Nal. Points are shown as percentage of colony formation without Nal (104.7 ± 4.0 per 1,000) in soft agar after culture in the absence of Nal.

substratum and were easily detached from dishes after confluence in the absence of Nal. The dose–response curve for the inhibition of colony formation of TF-2 cells on a collagen-coated dish by various concentrations of Nal was similar to that on a solid support shown in Figure 1 (data not shown). As shown in Figure 5, the saturation density of TF-2 cells in the presence of 700 μM Nal was about 50% lower than that without Nal. Since the saturation density of non-transformed Balb/3T3 cells was about $3.5 \times 10^6$, the observed saturation density of TF-2 cells in the presence of 700 μM Nal was still about 60% higher than that of non-transformed cells. In parallel with the cell growth, a typical example of morphological change at the confluent state induced by Nal (700 μM) was shown in Figure 6. The irregular arrangement of the cells in the absence of Nal contrasts with the more ordered arrangement in the presence of Nal. This morphological change was visible even 24 h after

**Figure 5** Effects of Nal on growth of TF-2 cells. The cells were seeded at $1.0 \times 10^6$ per 60 mm collagen coated Petri dish. 24 h after seeding different concentrations of Nal were added to the cultures. Symbols: ○, 0 μM Nal; ▲, 500 μM; ■, 700 μM; □, Nal was removed on the third day of culture.

**Figure 6** Representative morphological change of TF-2 cells during growth after confluence. Cells were cultured as shown in Figure 5 and were photographed on the third day of culture. **a**, 0 μM Nal; **b**, 700 μM.

addition of Nal. The lower saturation density of treated cells increased to higher saturation density after removal of Nal. Thus, we concluded that Nal reversibly restored density-dependent growth control for transformed cells as well as anchorage-dependent growth.

### *Comparison of effects of Nal derivatives on suppression of growth in soft agar*

The difference between the growths of cells in soft agar and on a solid support is an index of the activity of a test compound to suppress anchorage-independent growth of transformed cells preferentially. Figure 7 shows the dose–response curves of colony formation in soft agar and on a solid support for Oxl and PPA. The $SD_{50}/AD_{50}$ ratios for Oxl and PPA were estimated to be 3.0 and 1.4 (a higher $SD_{50}/AD_{50}$ value indicates that the compound has a greater suppressive effect). Thus Oxl and PPA showed weak activity in this assay, although they did not alter the morphology of transformed foci. Judging from their $SD_{50}/AD_{50}$ values the activities of the three 4-quinolones were in the following order: Nal > Oxl > PPA.

### *Effects of Nal on NIH/3T3 cells transformed by an activated c-Ha-ras*

Next we examined whether Nal suppressed anchorage-independent growth of cells transformed by only chemicals, or also, more generally, of those transformed by oncogenes. For this purpose, we used NIH/3T3 cells (F25), which were transformed by an activated c-Ha-*ras* gene. Figure 8 shows the dose–response curves for Nal of colony formation of F25 cells in soft agar and on a solid support. The growth of F25 cells on a solid support was slightly more sensitive to Nal than that of TF-2 cells, but growth of F25 cells in soft agar was suppressed dose-dependently by lower concentrations of

**Figure 7** Colony formation of TF-2 cells on a solid support (open symbols) and in soft agar (closed symbols) in the presence of various concentrations of Oxl (**a**) or PPA (**b**). The $SD_{50}$ and the $AD_{50}$ values for Oxl were 330 and 110 μM (means of three and two experiments), respectively, and those for PPA were 130 and 90 μM (means of three experiments), respectively.

Nal than those required to suppress growth on a solid support: the $SD_{50}/AD_{50}$ ratio was 3.2. Thus, Nal preferentially suppressed anchorage-independent growth of transformed cells induced by either chemicals or an oncogene.

Since Nal inhibited preferentially the growth of F25 cells in soft agar compared to that on a solid support, it was important to examine whether Nal affected the amount of p21 in

**Figure 8** Colony formation of activated c-Ha-*ras*-transformed mouse cells (F-25) on a solid support (open symbols) and in soft agar (closed symbols) in the presence of various concentrations of Nal. The $SD_{50}$ and $AD_{50}$ values were 590 and 180 μM (means of three and two experiments), respectively.

**Figure 9** Detection of p21 in NIH/3T3 cells and F25 cells by immunoblotting analysis using the monoclonal antibody, NCC-RAS-004. F25 cells ($2 \times 10^5$ per 60 mm dish) were cultured in DMEM/F12 containing 10% FCS and different concentrations of Nal for 3 days. NIH/3T3 cells were cultured without Nal as a negative control. Preparation of cell lysates and methods of analysis are described in Materials and methods. Lane 1, NIH/3T3 cells; lane 2, F25 cells without Nal treatment; lane 3, F25 cells treated with 150 μM; lane 4, F25 cells treated with 300 μM Nal.

(data not shown). Thus, the preferential inhibition of growth in soft agar by Nal was not due to a reduction in the amount of p21.

### Discussion

In this work, we show that Nal reversibly suppressed transformed phenotypes of mouse cells induced by either 3-methylcholanthrene or an activated c-Ha-*ras* gene. The following observations are mutually consistent with this conclusion. First, Nal preferentially suppressed the growth in soft agar of transformed Balb/3T3 mouse cells induced by either MC or by an activated c-Ha-*ras* gene (Figures 1 and 8), without affecting the amount of p21 for the latter. The preferential suppression of growth in soft agar was concluded to be due to the effect on anchorage-independent growth, because agar did not induce additional cytotoxicity by Nal on a solid support (Figure 1) and Nal effectively inhibited colony growth in soft agar but not growth on a solid support (Figure 2). Second, the saturation density of growth of MC-induced transformed cells was reduced by the presence of Nal. In the exponentially growing state and at an early F25. The amount of p21 in F25 cells at the late log phase was measured by use of a monoclonal antibody against the protein. Since the difference in the staining cannot be regarded as significant, as shown in Figure 9, we concluded that the amount of the protein was not changed in the presence of 150 and 300 μM Nal. The amount of the protein was not changed even for cells harvested at a confluent state confluent state, transformed cells grew at a similar rate irrespectively of the presence of Nal (Kaneko & Horikoshi, 1988). However, after confluence, the saturation density of transformed cells was reduced in the presence of Nal. In parallel with the reduced growth rate after confluence, the morphology of transformed cells was changed to a more ordered arrangement (Figure 6) in the presence of Nal. Consistent with this observation, the number of morphologically transformed foci which had been induced by MC was reduced in the presence of Nal (data not shown). Third, the suppressive effects of Nal on both the growth in soft agar and the growth at reduced saturation density were reversible. Since no difference was found between the colony formation in soft agar of cells previously exposed to Nal (500 μM) for 2 months (data not shown) and that of cells exposed to Nal for only 6 days (Figure 4), Nal induced possibly only a limited reversion from the transformed state. The idea is supported by the fact that morphological change started even after incubation for 24 h in the presence of Nal. Finally, Oxl and PPA, which were derivatives of Nal, were less effective than Nal in suppressing growth in soft agar. Previously, we found that Nal inhibited both the early and the later stage of MC-induced morphological transformation. These findings, together with the present results, show that Nal reversibly inhibits the expression of transformed phenotypes at all stages during the process of neoplastic transformation of mouse cells, and also after the transformed phenotypes are expressed.

In the case of oncogene-induced transformation of cells, a general model for origins of revertant cells was proposed by Bassin and Noda (1987). According to their model, revertants are subdivided into oncoprotein-related and target (of the oncoprotein)-related types: the former revertants arise by the loss or inactivation of a transformed gene, whereas the latter continue to express the transforming protein, but are phenotypically normal or quasi-normal. This classification can be applied to aparent revertants induced by the presence of chemicals (referred to as 'chemical suppressors'). Ansamycins, chemical suppressors of anchorage-independent growth of cells transformed by an oncogene (v-*src*), inhibit phosphorylation of the *src* gene product $pp60^{src}$ (Murakami *et al.*, 1988). Thus, ansamycins induce oncoprotein-related revertant cells. The effects of retinoids on neoplastic transformation *in vitro* and *in vivo*, as well as on differentiation, have been studied extensively (Roberts & Sporn, 1984), but they have been found to vary depending on the cells used: retinoids suppress anchorage-independent growth of transformed cells (Roberts & Sporn, 1984; Levine *et al.*, 1986) and change the morphology of chemically transformed BALB/3T3 cells (Yamasaki & Katoh, 1988), whereas they do not inhibit growth of 10T1/2 mouse cells transformed by oncogenes (BJ-*ras*, v-*ras*; Bertram & Martner, 1985) or by methylcholanthrene (Merriman & Bertram, 1979), and even enhance transformation of mouse epidermal cells (Kulesz-Martin *et al.*, 1986). Thus, retinoids are considered to have different effects depending on the cell type and so cannot be classified simply in the above way. As Nal suppressed anchorage-independent growth of cells transformed by either MC or activated c-Ha-*ras* and Nal did not inhibit the production of p21, it seems to inhibit some step that is common to both types of transformation and that controls anchorage-independent growth. Thus, Nal must induce the target-related type of reversion.

Nal, Oxl and PPA differed in ability to suppress anchorage-independent growth preferentially, as judged by their $SD_{50}/AD_{50}$ ratio. Use of this $SD_{50}/AD_{50}$ ratio as an index of activity eliminates the influence of difference in permeability of these compounds on observed results. Nal and Oxl have been reported to inhibit DNA topoisomerase II from Hela cells (Miller *et al.*, 1981) and *Drosophila* (Shelton *et al.*, 1983) as well as bacterial DNA gyrase (Domagala *et al.*, 1986). Although these compounds have not been shown to act specifically on topoisomerase II, we previously presented a hypothesis that DNA supercoiling may stimulate certain genes that play a role in morphological transformation and tht Nal and Oxl may suppress the expression of these genes, modulating the activity of topoisomerase II (Kaneko & Horikoshi, 1988). This hypothesis was based on the observation that DNA supercoiling stimulates *in vitro* transcription of the fibroin gene and adeno virus 2 major late promoter, but not that of the *hsp* 70 gene (Hirose & Suzuki, 1988). This hypothesis is supported by the recent finding that novobiocin, a DNA topoisomerase inhibitor, inhibits SV 40 enhancer activity (Kohno *et al.*, 1988). The present results may allow extension of this hypothesis to control of genes already expressed. As the $SD_{50}/AD_{50}$ ratio was not the same for all three 4-quinolones, the preferential suppression of anchorage-independent growth cannot be explained only by modulating effects of topoisomerase II. The structure of the ring adjacent to the common 4-pyridone ring of these compounds may modify their toxicity and their effect on growth in soft agar to different extents.

Studies are required on the precise relationship between the chemical structures of 4-quinolones and their suppressive activities with a view to obtaining compounds more effective than Nal. In addition, further studies on the effects of Nal on anchorage-independent growth of various transformed cells and on the expression of various oncogenes should provide information both on the mechanism controlling anchorage-independent growth and on the target of Nal.

We are grateful to Dr T. Sekiya for the gift of F25 and NIH/3T3 cells, to Dr S. Hirohashi for the gift of a monoclonal antibody, NCC-RAS-004, to Drs A. Hoshi and M. Inomata for advice in the culture in soft agar, to Drs M. Kodama and T. Kimura for valuable comments on the manuscript, and to Dai-nihonseiyaku Co. for the gift of pipemidic acid.

### References

BASSIN, R.H. & NODA, M. (1987). Oncogene inhibition by cellular genes. *Adv. Viral Oncol.*, **6**, 103.

BERTRAM, J.S. & MARTNER, J.E. (1985). Inhibition by retinoids of neoplastic transformation *in vitro*: cellular and biochemical mechanisms, In *Retinoids, Differentiation and Disease*, Ciba Foundation Symposium 113, p. 29. Pitman: London.

DAVIS, L.G., DIBNER, M.D. & BATTEY, J.F. (1986). *Basic Methods in Molecular Biology*. Elsevier: New York.

DER, C.J. & COOPER, G.M. (1983). Altered gene products are associated with activation of cellular $ras^k$ genes in human lung and colon carcinomas. *Cell*, **32**, 201.

DOMAGALA, J.M., HANNA, L.D., HEIFETZ, C.L. & 4 others (1986). New structure- activity relationships of the quinolone antibacterials using the target enzyme. The development and application of a DNA gyrase activity. *J. Med. Chem.*, **29**, 394.

FERRAZZI, E., PERACCHI, M., BIASOLO, M.A. & 3 others (1988). Antiviral activity of gyrase inhibitors norfloxacin, coumermycin $A_1$ and nalidixic acid. *Biochem. Pharmacol.*, **37**, 1885.

GALLAGHER, M., WEINBERG, R. & SIMPSON, M.V. (1986). Effect of the bacterial DNA gyrase inhibitors, novobiocin, nalidixic acid, and oxolinic acid, on oxidative phosphorylation. *J. Biol. Chem.*, **261**, 8604.

HIROSE, H. & SUZUKI, Y. (1988). *In vitro* transcription of eukaryotic genes is affected differently by the degree of DNA supercoiling. *Proc. Natl Acad. Sci. USA*, **85**, 718.

IKEDA, S., YAZAWA, M. & NISHIMURA, C. (1987). Antiviral activity and inhibition of topoisomerase by ofloxacin, a new quinolone derivative. *Antiviral Res.*, **8**, 103.

INOMATA, M., KANEKO, A. & HOSHI, A. (1986). Improved colony formation of cultured retinoblastoma cells. *Invest. Ophthalmol. Vis. Sci.*, **27**, 1423.

KANAI, T., HIROHASHI, S., NOGUCHI, M. & 5 others (1987). Monoclonal antibody highly sensitive for the detection of ras p21 in immunoblotting analysis. *Jpn. J. Cancer Res. (Gann)*, **78**, 1314.

KANEKO, M. & HORIKOSHI, J. (1987). Topoisomerase inhibitors suppressed lithocholic acid-induced promotion of transformation in BALB/3T3. *Br. J. Cancer*, **56**, 614.

KANEKO, M. & HORIKOSHI, J. (1988). Nalidixic acid and oxolinic acid reversibly suppress 3-methylcholanthrene-induced cell transformation of BALB/3T3 mouse cells. *Int. J. Cancer*, **42**, 913.

KOHNO, K., IWAMOTO, Y., MARTIN, G.R. & 1 other (1988). Novobiocin inhibits the SV40 enhancer activity. *Biochem. Biophys. Res. Commun.*, **154**, 483.

KULESZ-MARTIN, M., BLUMENSON, L. & LISAFELD, B. (1986). Retinoic acid enhancement of an early step in the transformation of mouse epidermal cells *in vitro*. *Carcinogenesis*, **7**, 1425.

LAEMML, U.K. (1970). Cleavage of structural proteins during the assembly of the head of bacteriophage T4. *Nature*, **227**, 680.

LEVINE, A.E., CRANDALL, C.A., BRATTAIN, D. & 2 others (1986). Retinoic acids restores normal growth control to a transformed mouse embryo fibroblast cell line. *Cancer Lett.*, **33**, 33.

MERRIMAN, R.L. & BERTRAM, J.S. (1979). Reversible inhibition by retinoids of 3-methylcholanthrene-induced neoplastic transformation in C3H/10T1/2 clone 8 cells. *Cancer Res.*, **39**, 1661.

MILLER, K.G., LIU, L.F. & ENGLUND, P.T. (1981). A homogenous type II DNA topoisomerase from HeLa cell nuclei. *J. Biol. Chem.*, **256**, 9334.

MURAKAMI, Y., MIZUNO, S., HORI, M. & 1 other (1988). Reversal of transformed phenotypes by herbimycin A in *src* oncogene expressed rat fibroblasts. *Cancer Res.*, **48**, 1587.

ROBERTS, A.B. & SPORN, M.B. (1984). Cellular biology and biochemistry of the retinoids. In *The Retinoids, vol. 2*, Sporn, M.B., Roberts, A.B. & Goodman, D.S. (eds) p. 209. Academic Press: New York.

SEKIYA, T., PRASSOLOV, V.S., FUSHIMI, M. & 1 other (1985). Transforming activity of the c-Ha-*ras* oncogene having two point mutations in codons 12 and 61. *Jpn. J. Cancer Res. (Gann)*, **76**, 851.

SHELTON, E.R., OSHEROFF, N. & BRUTLAG, D.L. (1983). DNA topoisomerase II from *Drosophila melanogaster*; relaxation of supercoiled DNA. *J. Biol. Chem.*, **258**, 9536.

SHIN, S.I., FREEDMAN, V.H., RISSER, R. & 1 other (1975). Tumorgenicity of virus-transformed cells in nude mice is correlated specially with anchorage independent growth *in vitro*. *Proc. Natl Acad. Sci. USA*, **72**, 4435.

STANBRIDGE, E.J., CHANNING, J.D., DOERSEN, C.-J. & 4 others (1982). Human cell hybrids: analysis of transformation and tumorgenicity. *Science*, **215**, 252.

YAMASAKI, H. & KATOH, F. (1988). Further evidence for the involvement of gap-junctional intercellular communication in induction and maintenance of transformed foci in BALB/3T3 cells. *Cancer Res.*, **48**, 3490.